# Rho/MRTF-A-Induced Integrin Expression Regulates Angiogenesis in Differentiated Multipotent Mesenchymal Stem Cells

**DOI:** 10.1155/2015/534758

**Published:** 2015-04-08

**Authors:** Rui Zhang, Nan Wang, Man Zhang, Li-Nan Zhang, Zhi-Xia Guo, Xue-Gang Luo, Hao Zhou, Hong-Peng He, Tong-Cun Zhang

**Affiliations:** ^1^Key Laboratory of Industrial Microbiology, Ministry of Education and Tianjin City, College of Biotechnology, Tianjin University of Science and Technology, Tianjin 300457, China; ^2^Department of Biochemistry, Medical College, Wuhan University of Science and Technology, Wuhan 430081, China

## Abstract

Mesenchymal stem cells (MSCs) are known to undergo endothelial differentiation in response to treatment with vascular endothelial growth factor (VEGF), but their angiogenic ability is poorly characterized. In the present study, we aimed to further investigate the role of Rho/MRTF-A in angiogenesis by MSCs and the effect of the Rho/MRTF-A pathway on the expression of integrins *α*1*β*1 and *α*5*β*1, which are known to mediate physiological and pathological angiogenesis. Our results showed that increased expression of *α*1, *α*5, and *β*1 was observed during angiogenesis of differentiated MSCs, and the Rho/MRTF-A signaling pathway was demonstrated to be involved in regulating the expression of integrins *α*1, *α*5, and *β*1. Luciferase reporter assay and ChIP assay determined that MRTF-A could bind to and transactivate the integrin *α*1 and *α*5 promoters. Treatment with the Rho inhibitor C3 transferase, the Rho-associated protein kinase (ROCK) inhibitor Y27632 or with shMRTF-A inhibited both the upregulation of *α*1, *α*5, and *β*1 as well as angiogenesis. Furthermore, in human umbilical vein endothelial cells (HUVECs), MRTF-A deletion led to marked reductions in cell migration and vessel network formation compared with the control. These data demonstrate that Rho/MRTF-A signaling is an important mediator that controls integrin gene expression during MSC-mediated angiogenic processes.

## 1. Introduction

Mesenchymal stem cells (MSCs) can mediate postnatal neovascularization and angiogenesis by multiple mechanisms, including the indirect secretion of angiogenic factors [1, 2] and in situ differentiation into endothelial cells (ECs) [3]. In response to blood vessel injury, MSCs residing in the bone marrow can be mobilized and access the circulation, where they then contribute to cell replacement and neovascularization in vivo [2]. Others have shown that MSCs can differentiate into endothelial cells in vitro, and the potential molecular mechanism governing endothelial differentiation has subsequently been investigated [4, 5]. However, the regulatory mechanism underlying MSC-mediated angiogenesis is still unclear.

Integrins are a family of transmembrane glycoproteins consisting of noncovalently linked *α* and *β* chains that mediate cell-cell and cell-matrix interactions [6]. Many integrins, including *α*1*β*1 and *α*5*β*1, mediate physiological angiogenesis in addition to being involved in regulating angiogenesis under pathological conditions. Integrin *α*5*β*1 is upregulated in blood vessels in human tumor biopsies and plays a critical role in angiogenesis, promoting tumor growth in vivo [7]. Integrin *α*1*β*1 is thought to be proangiogenic, as either functional blocking antibodies or targeted deletion of the *α*1 subunit results in a decrease in both vascular endothelial growth factor-mediated as well as tumor-associated angiogenesis [8]. In preclinical studies, pharmacological inhibition of integrin function efficiently suppressed angiogenesis and inhibited tumor progression. Integrins *α*1*β*1 and *α*5*β*1 might be potential therapeutic targets for the suppression of tumor angiogenesis [9]. Therefore, in the present study we aimed to further investigate the role of integrins during MSC-mediated angiogenesis.

Our previous studies reported that Rho/MRTF-A is essential for the VEGF-induced endothelial differentiation of MSCs. It has been demonstrated that Rho/Rho-associated protein kinase (ROCK) signaling is an important mediator in a number of angiogenic processes, including EC migration, survival, and cell permeability, suggesting that Rho/ROCK inhibition may prove useful for the treatment of angiogenesis-related disorders [10]. Rho signaling is reported to be essential for VEGF-dependent in vivo angiogenesis and in vitro capillary formation [11]. Upon the activation of Rho/ROCK signaling, fibrillar actin (F-actin) accumulation leads to the depletion of G-actin and the release of MRTF-A, which then translocates to the nucleus and promotes the transcription of target genes [12]. Thus, we asked whether MRTF-A might play an important role not only in endothelial differentiation but also in MSC-mediated angiogenesis. Specifically, we examined whether MRTF-A mediates angiogenesis through the transcriptional regulation of integrin target genes.

Here, we identify and characterize MRTF-A as a regulator of differentiated-MSC angiogenesis. MRTF-A knockdown partially inhibits the differentiation of MSCs into endothelial cells and strongly abrogates VEGF-induced vessel outgrowth. Increased expression of integrins *α*1, *α*5, and *β*1 was observed in MSCs during endothelial cell differentiation, whereas treatment with the Rho inhibitor C3 transferase, the ROCK inhibitor Y27632 or with siMRTF-A inhibited the upregulation of integrin expression. Moreover, reporter assays and ChIP assay demonstrated that MRTF-A bound to and transactivated the integrin *α*1 and *α*5 promoters.

## 2. Materials and Methods

### 2.1. Cell Culture and Treatment

Rat bone marrow-derived MSCs were isolated from the femurs and tibias of male Sprague-Dawley rats (weight 90–100 g) as described previously. Sprague-Dawley rats (male, 4–6 weeks old) were obtained from the Laboratory Animal Center of the PLA, Academy of Military Medical Sciences (Beijing, China). All animal procedures were approved by the institutional animal and care use committee of Tianjin University of Science & Technology. The cells were seeded in Dulbecco's modified eagle's medium-low glucose (DMEM-LG, Hyclone) supplemented with 10% Fetal Bovine Serum (FBS, PAA) at 37°C in a humidified incubator with a 5% CO_2_ atmosphere. MSCs were phenotypically characterized according to the method published by Wang et al. [13]. Flow cytometric analyses showed that the MSC cultures represented a homogeneous cell population devoid of hematopoietic cells. COS-7 cells and human umbilical vascular endothelial cells (HUVECs) were kindly provided by Tianjin Medical University.

To assess endothelial differentiation potential, MSCs were cultured in basal DMEM-LG medium supplemented with 50 ng/mL VEGF (AF-100-20, Peprotech) and 2% FBS for 7 d. Medium was changed every 2 d.

C3 cell permeable transferase is a specific RhoA inhibitor [14]. Y-27632 is a pharmacological inhibitor of ROCK that competitively inhibits the ATP-binding domain of ROCK1 and ROCK2 [15]. Here, the cells were treated with 2 *μ*g/mL C3 transferase or 10 *μ*mol/L Y27632.

### 2.2. MTT Assay

Cell viability and proliferation were examined by 3-(4,5-dimethylthiazol-2-yl)-2,5-diphenyltetrazolium (MTT) assay (Sigma). The absorbance of each well was measured using a Synergy 4 plate reader (Bioteck) with a wavelength of 490 nm, with the reference wavelength set at 630 nm. Absorbance was directly proportional to the number of survival cells.

### 2.3. Uptake of Acetylated Low-Density Lipoprotein (DiI-Ac-LDL)

Acetylated low-density lipoprotein (DiI-Ac-LDL; Biomedical Technologies Inc., MA) was diluted to 10 *μ*g/mL in complete growth medium and added to the cells and incubated at 37°C. The medium was then removed, and the cells were washed with PBS. Cells were fixed with 4% paraformaldehyde and DiI-Ac-LDL uptake (Red) was visualized using a laser scanning confocal microscope (OLYMPUS).

### 2.4. Matrigel In Vitro Angiogenesis Assay

MSCs were cultured in endothelial differentiation medium for 7 d and capillary tube formation was then induced using an in vitro angiogenesis kit (Chemicon). Briefly, basement membrane-like material (EC Matrix; BD) was diluted to 0.5–0.7 mg/mL in endothelial differentiation medium. A total of 5 × 10^4^ cells were seeded in 300 *μ*L of 0.5–0.7 mg/mL Matrigel in each well of a 24-well plate. The Matrigel-cell suspension was polymerized for 4 h at 37°C. Then, 300 *μ*L of endothelial differentiation medium supplemented with 50 ng/mL VEGF was added, and the gel-embedded cells were cultured at 37°C and 4% CO_2_. The structures were photographed using a phase contrast microscope (Olympus). Total cord length was quantified using image-Pro Plus v4.5 software.

### 2.5. Quantitative Real-Time Polymerase Chain Reaction (RT-PCR)

Total RNA was extracted from cells using TRIzol reagent (Invitrogen), and 2 *μ*g of RNA was used as a template for reverse transcription using random primers. qRT-PCR was performed to characterize the mRNA levels of specific genes using Fast SYBR Green Master Mix (Applied Biosystems) in a Biosystems StepOne Real-Time PCR machine (Applied Biosystem, CA). Glyceraldehyde-3-phosphate dehydrogenase (GAPDH) expression was used for normalization. The primers used in real-time PCR experiments were as follows: integrin *α*1 F-GCT­GGC­TCC­TCA­CTG­TTG­TT, R-CTC­CAT­TTG­GGT­TGG­TGA­CT; integrin *α*5 F-CAT­AAG­ACA­GAT­AGC­ACC­CG, R-TCC­TAC­CTG­CCC­TAA­CGT; integrin *β*1 F-TGG­ACG­AAA­GTG­CTC­TAA­CA, R-GAA­CTG­AAG­GAC­CAC­CTC­TAC.

### 2.6. Western Blotting

Western blotting was performed according to a standard method, as described previously [13]. The following primary antibodies were used: rabbit anti-integrin *α*5 (Bioss), anti-integrin *α*1 (Bioss), anti-integrin *β*1 (Abcam), and mouse anti-GAPDH (Santa Cruz). Antibody incubations were performed overnight at 4°C. The secondary antibodies used were IRDye-800-conjugated anti-mouse and anti-rabbit IgG (Li-COR Biosciences). Immunoreactivity was detected using an Odyssey Infrared Imaging System (Gene Company Limited). The relative quantification of the protein expression was statistically analyzed using Image J software.

### 2.7. siRNA Transfection

For short-term depletion experiments, the following siRNA sequences were used: GAA­UGU­GCU­ACA­GUU­GAA­A, human and rat MRTF-A (siMRTF-A) (Invitrogen); UUC­UCC­GAA­CGU­GUC­ACG­U, control siRNA (Invitrogen). MSCs and HUVECs were transfected with siRNA oligonucleotides using Lipofectamine 2000 (Invitrogen) according to the manufacturer's instructions. Cell migration assay, transwell chamber assay, PCR and western blotting were performed as described.

### 2.8. Cell Migration Assay

Differentiated MSCs and HUVECs grown in 6-well plates were transfected with siMRTF-A or control siRNA in the presence of VEGF and then wounded using a sterile pipette tip to remove cells. The progress of migration was photographed immediately following injury, and micrographs were taken after zero, 24, and 48 h.

### 2.9. Transwell Chamber Assay

After differentiated MSCs and HUVECs were transfected with siMRTF-A or control siRNA, cells were harvested by trypsin, and 1.0 × 10^4^ cells in 200 *μ*L of 1% FBS-containing medium were then seeded into the upper chamber of a Transwell cell culture insert. The lower chamber was filled with 600 *μ*L of medium containing 10% FBS. Twenty-four hours later, the cells in the upper chamber were removed using a cotton swab. Cells that had migrated to the lower side of the membrane were fixed with 4% paraformaldehyde and stained with DAPI. The number of migrated cells was counted and photographed in five fields (the upper, lower, left, right, and middle) in three independent experiments.

### 2.10. Plasmids, Cell Transfection, and Luciferase Assays

The pcDNA3.1-MRTF-A, shRNA-MRTF-A (sh-MRTF-A), and pSUPER (control) plasmids used in this study have been reported previously [13]. pSUPER was a negative control plasmid consisting of a circle plasmid encoding a siRNA whose sequence was not found in mouse, human, or rat genome databases. The integrin *α*1 promoter region (−612/+210) and the integrin *α*5 promoter region (−1644/+46) were each amplified by PCR and then cloned into a pGL3 luciferase reporter vector. The primers used to create integrin *α*1-luc and integrin *α*5-luc were as follows: integrin *α*1 F-CAC­GGG­TAC­CTT­ATT­AAC­CAG­AAG­CAG­G, R-GCT­CAA­GCT­TAG­GCA­TCC­TCC­TCC­CAT­A; integrin *α*5 F-TGG­TAC­GCG­TAC­TTG­CTA­CCT­CAA­ACC­T, R-CCT­TTC­CAG­ATG­TAC­AAC­CTC­GAG­GCT­C.

Transfection reporter assays were performed in 24-well plates. MSCs, HUVECs and COS-7 cells were cultured in growth medium without antibiotics at 60% confluency for 2 d. MSCs were then transfected using the FuGENE HD transfection reagent (Roche); HUVECs and COS-7 cells were transfected using the TurboFect transfection reagent (Thermo). For each transfection, 0.2 *μ*g of reporter was used in combination with the expression plasmids encoding MRTF-A (0.8, 1, or 1.2 *μ*g). After incubation for 6 h, the medium was removed and replaced with normal culture medium. Cells were harvested 24 h after transfection, and luciferase activity was measured using the Dual luciferase Assay system (Promega). The results were normalized by dividing the Firefly luciferase activity with the Renilla luciferase activity of the same sample.

### 2.11. ChIP Assay

ChIP analysis was performed using a commercially available kit (Enzymatic Chromatin IP (Magnetic Beads), Cell Signaling Technology) in MSCs treated with VEGF at d2, d4 and d7. Proteins were cross-linked to DNA by formaldehyde at a final concentration of 1% for 20 min at room temperature. Protein-DNA complexes were immunoprecipitated using primary antibodies for MRTF-A (Sigma). The signal of MRTF-A/integrin *α*1 promoter complexes and MRTF-A/integrin *α*5 promoter complexes were measured by PCR. The primers used for amplification of integrin *α*1 and integrin *α*5 promoter were as followed: integrin *α*1 F-CAG­GTT­GCG­TAG­CCA­TCC, R-TGG­TAG­CCA­CCT­GCC­TCT; integrin *α*5 F-GAG­AAA­AGG­GAG­GCT­AGG, R-ACA­CAG­GAG­CCC­CTT­AGT.

### 2.12. Statistical Analysis

Data were expressed as the mean ± SE, accompanied by the number of experiments performed independently and analyzed by *t*-test. Differences with *P* < 0.05 were considered statistically significant.

## 3. Results

### 3.1. VEGF Induces Angiogenesis and High Integrin Expression in Differentiated MSCs

**Figure 1:**
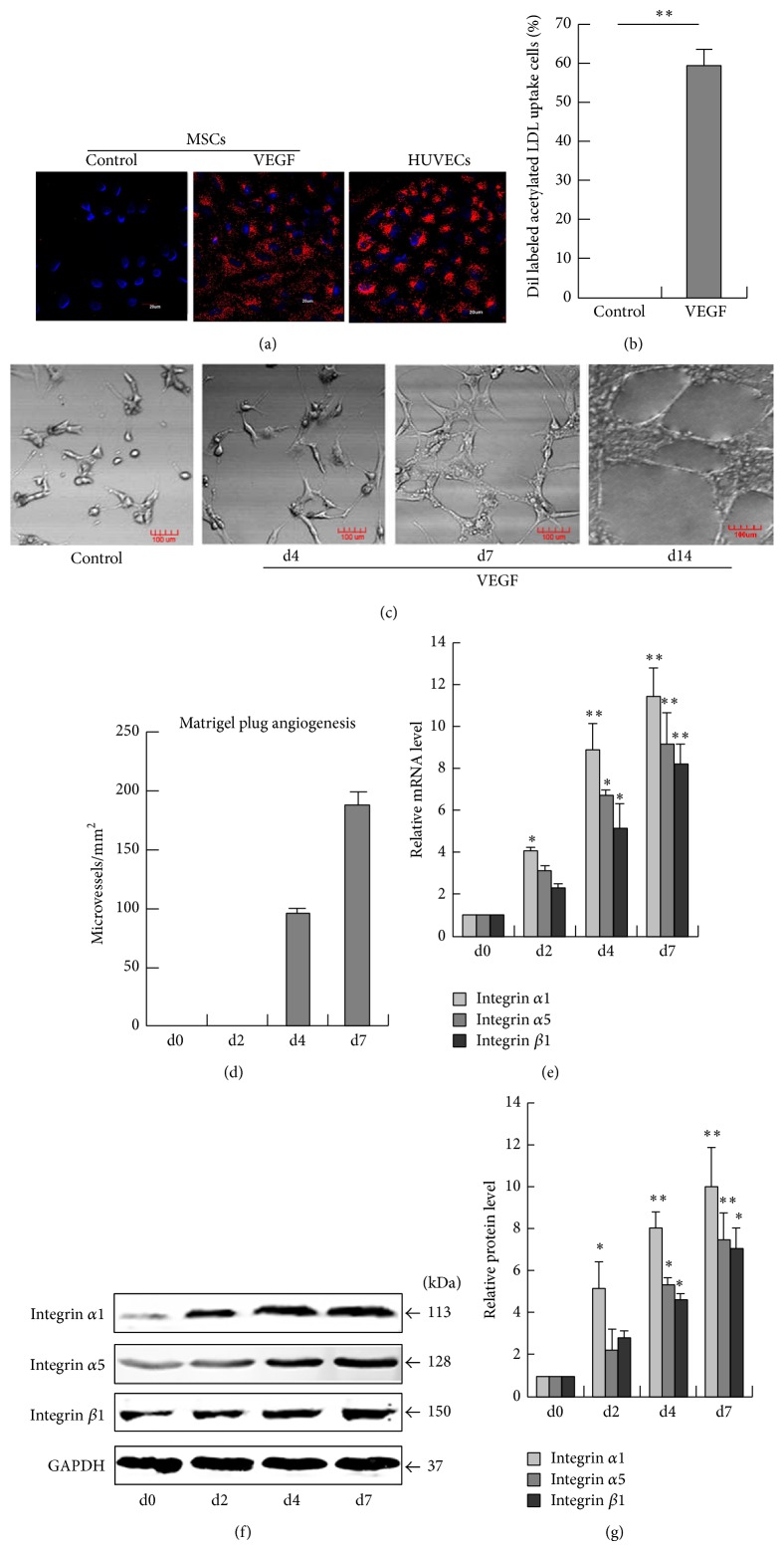
VEGF induces angiogenesis and high integrin expression in differentiated MSCs. (a) DiI-Ac-LDL uptake assay in differentiated MSCs. Parallel experiments in HUVECs served as a positive control. (b) Percentages of MSCs that incorporated DiI-Ac-LDL were calculated, and the data were expressed as percentages compared with the overall cell count. ^∗∗^ *P* < 0.01, *n* = 3. (c) MSCs seeded onto a basement membrane-like gel were treated with VEGF for 4, 7, and 14 days. VEGF-free MSCs were seeded onto a basement membrane-like gel for 7 days (Control group). (d) Capillary-like structures were quantified by measuring the polygonal network. (e) After MSCs were cultured in EC differentiation medium for 7 d, integrins *α*1, *α*5, and *β*1 were examined by qPCR. ^∗^ *P* < 0.05; ^∗∗^ *P* < 0.01, *n* = 3. (f) Western blotting assay for integrins *α*1, *α*5, and *β*1. (g) The relative quantification of the protein expression was statistically analyzed using Image J software. ^∗^ *P* < 0.05; ^∗∗^ *P* < 0.01, *n* = 3.

DiI-Ac-LDL uptake was analyzed to assess the functionality of the differentiated cells. Our results showed that these VEGF-differentiated cells acquired the ability to incorporate DiI-Ac-LDL (Figures 1(a) and 1(b)). Parallel experiments in HUVECs served as a positive control. To assess angiogenic capacity, MSCs were seeded onto a basement membrane-like gel with VEGF for 4, 7 and 14 days. As shown in Figure 1(c), the MSCs untreated with VEGF mostly remained rounded. VEGF-treated MSCs start to form capillary-like structures on Matrigel at d7. A stable and mature capillary-like structure was observed when MSCs were seeded on Matrigel at d14. Capillary-like structures were quantified by measuring the polygonal network at day 7 and 14, and a significant increase in the total tube area and branching points was observed (Figure 1(d)).

To investigate whether VEGF-treated MSCs upregulate integrins *α*1, *α*5, and *β*1, we detected the expression of integrins *α*1, *α*5, and *β*1 in VEGF-treated MSCs by qRT-PCR and western blotting. As shown in Figures 1(e)–1(g), the expression of integrins *α*1, *α*5, and *β*1 was markedly enhanced in VEGF-treated MSCs.

### 3.2. MRTF-A Is Essential for the VEGF-Induced Angiogenesis of Differentiated MSCs and Integrin *α*1, *α*5, and *β*1 Expression

**Figure 2:**
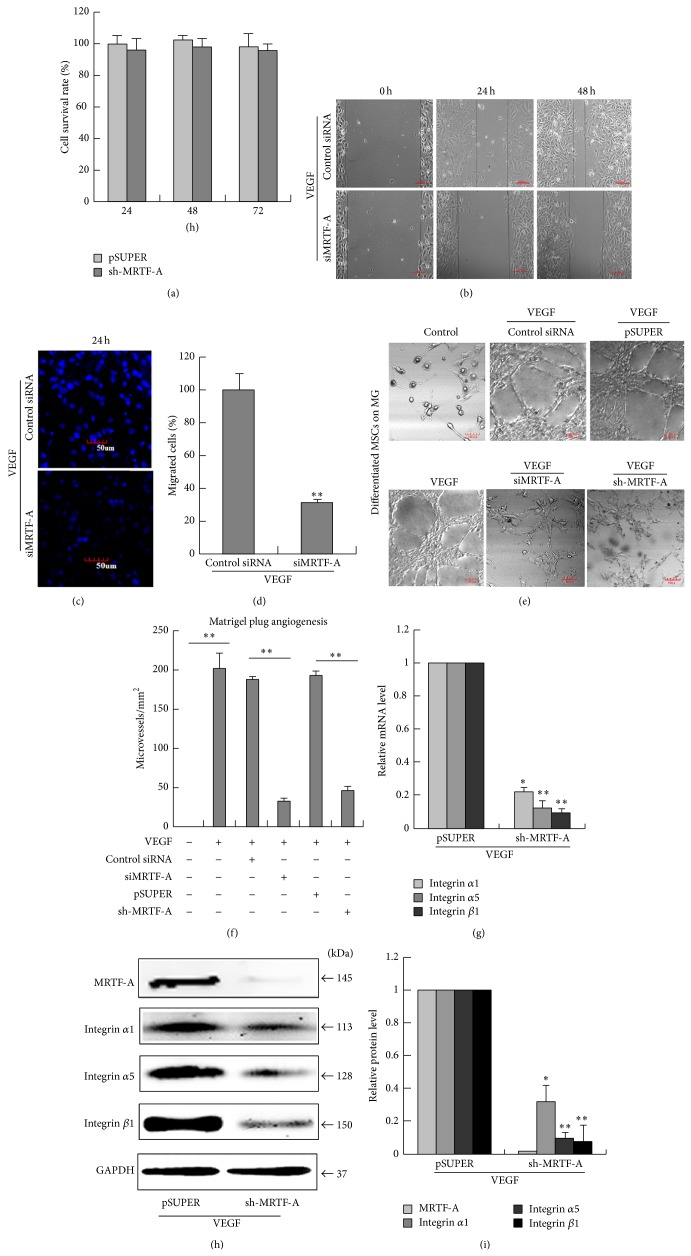
MRTF-A is essential for the VEGF-induced angiogenesis of differentiated MSCs and integrin *α*1, *α*5, and *β*1 expression. (a) After transfection with sh-MRTF-A or pSUPER (control), MSCs were cultured with EC differentiation medium for 3 d. MTT assay was performed to test the viability of MSCs. (b and c) MSCs were differentiated into ECs by treatment with VEGF for 7 d and siRNA-mediated knockdown experiments were then performed in the MSCs-derived ECs. The migratory ability of MSC-derived ECs transfected with si-MRTF-A or control siRNA was determined via wound healing (b) and transwell chamber assays (c). (d) Cell migration was quantified by calculating relative cell numbers. ^∗∗^ *P* < 0.01, *n* = 3. (e) After differentiation and siRNA or shRNA-mediated knockdown, the cells were cultured in matrigel for 7 d. Morphological changes in differentiated cells transfected with siMRTF-A or sh-MRTF-A were observed. (f) Capillary-like structures were quantified by measuring the polygonal network. ^∗∗^ *P* < 0.01, *n* = 3. (g) The expression of integrins *α*1, *α*5, and *β*1 was estimated by qPCR. ^∗^ *P* < 0.05; ^∗∗^ *P* < 0.01, *n* = 3. (h) The expression of integrins *α*1, *α*5 and *β*1 was estimated by western blot. (i) The relative quantification of the protein expression. ^∗^ *P* < 0.05; ^∗∗^ *P* < 0.01, *n* = 3.

MSCs transfected with sh-MRTF-A or pSUPER (control) were sequentially cultured in EC differentiation medium for 3 d, followed by MTT assay. Our results showed that MRTF-A knockdown did not affect cellular viability and proliferation (Figure 2(a)). To assess the role of MRTF-A in the migratory and angiogenic capacities of MSC-derived ECs, we performed siRNA or shRNA-mediated knockdown experiments in MSCs-derived ECs, followed by wound healing assays, transwell chamber assays and matrigel angiogenesis assays. After creating a “scratch” in a MSC monolayer, the images were captured at 0 h, 24 h and 48 h. As shown in Figure 2(b), the closure of preformed gaps in the cell layer was significantly decrease by siRNA against MRTF-A. For transwell chamber assays, these differentiated MSCs transfected with siMRTF-A or control siRNA were seeded into the upper chamber of a Transwell cell culture insert. After 24 hours of incubation under the cell culture conditions mentioned above, the cells that had crossed the monolayer and migrated to the lower side of the membrane were fixed, stained, photographed and counted with confocal scan microscope. There were significant migration and invasion in the control wells (control siRNA), whereas there was more than 60% reduction in the number of MRTF-A-knockdown cells that migrated across the transwell chamber membrane (Figures 2(c) and 2(d)). These results, as illustrated in Figures 2(b)–2(d), revealed that MRTF-A downregulation resulted in significant reduction in migration of MSCs-derived ECs. For the assessment of angiogenesis, VEGF-differentiated MSCs treated with siRNA or shRNA-mediated MRTF-A were seeded onto Matrigel in basic medium for 3 d. We found that differentiated MSCs formed capillary-like structure on Matrigel. MRTF-A-knockdown inhibited the formation of these structures in differentiated MSCs, which was demonstrated by a significant decrease in the total tube area and branching points (Figures 2(e) and 2(f)). These results confirmed that the VEGF-induced angiogenesis of MSCs may be dependent on MRTF-A.

To detect the effect of MRTF-A on the expression of integrins *α*1, *α*5, and *β*1, sh-MRTF-A-transfected MSCs were incubated with EC differentiation medium for 7 d and then the expression of integrins *α*1, *α*5 and *β*1 were tested by qPCR and western blotting. The results showed that the loss of MRTF-A function partly reduced the upregulation of integrins *α*1, *α*5 and *β*1 in MSCs stimulated with EC differentiation medium (Figures 2(g)–2(i)). These results confirmed that MRTF-A was a critical mediator of integrin *α*1, *α*5 and *β*1 expression in tube formation of differentiated MSCs.

### 3.3. MRTF-A Activates Integrin *α*1 and *α*5 Transcription by Binding to the Promoters of Integrin *α*1 and *α*5

**Figure 3:**
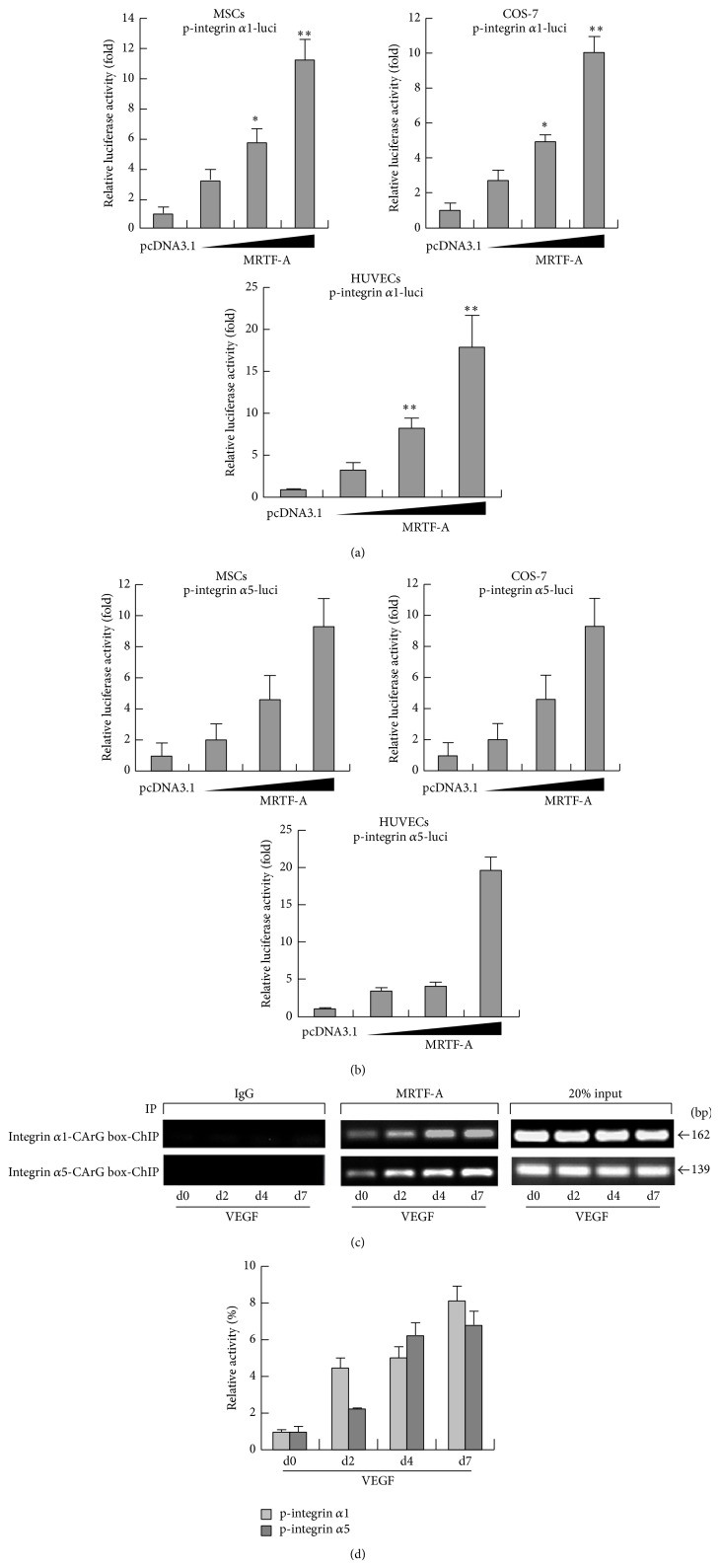
MRTF-A activates the transcription of integrins *α*1 and *α*5 by binding to the CArG box with their promoters. (a and b) Luciferase assays were performed 24 h after transfection of MRTF-A and integrin *α*1/integrin *α*5 promoter-luc plasmids into MSCs, COS-7 cells and HUVECs. ^∗^ *P* < 0.05; ^∗∗^ *P* < 0.01, *n* = 3. (c and d) ChIP assays were performed in MSCs treated with VEGF at d2, d4, and d7. Cross-linked chromatin was immunoprecipitated with specific anti-MRTF-A antibody. The precipitated chromatin DNA was then purified and amplified by reverse transcription PCR (c) and real time PCR (d) with specific primers that spanned CArG boxes in integrin promoters. The negative control in the immune-precipitation was performed with IgG antibody.

To determine whether MRTF-A can in fact activate the transcription of integrins *α*1 and *α*5, we constructed luciferase reporter gene constructs containing the integrin *α*1 and *α*5 promoters. Promoter-reporter assays showed that MRTF-A overexpression significantly increased the activities of the integrin *α*1 and *α*5 promoters in a dose-dependent manner in several cell types, including COS-7 cells, HUVECs and MSCs (Figures 3(a) and 3(b)). ChIP assays confirmed that VEGF induced recruitment of MRTF-A on the integrin *α*1 and *α*5 promoters in MSCs (Figures 3(c) and 3(d)). These data demonstrated that MRTF-A is a potent nuclear factor, which promoted the transcriptional activities of integrins.

### 3.4. VEGF-Induced Angiogenesis and Integrin Expression Depend on the Rho Signaling Pathway

**Figure 4:**
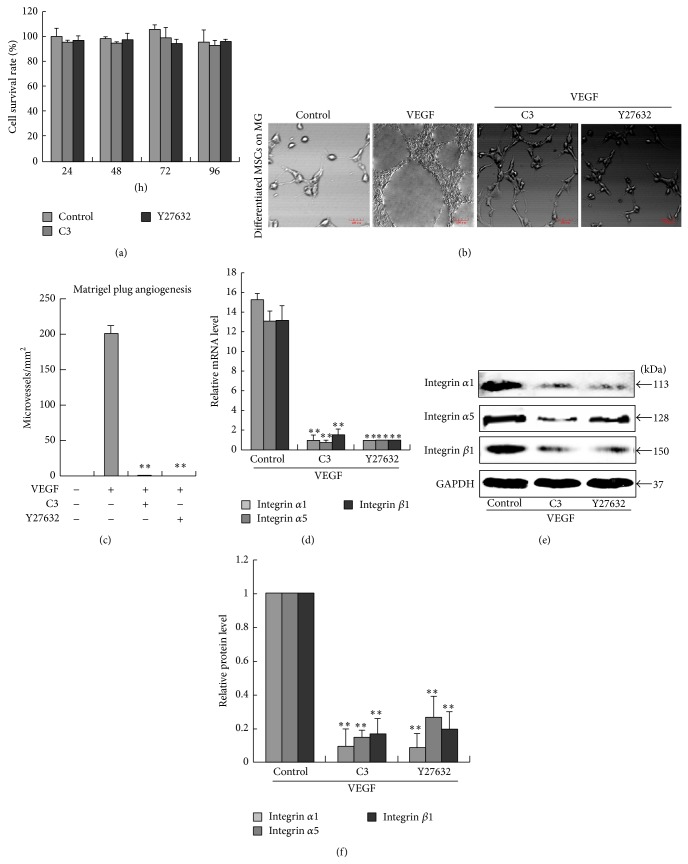
VEGF induces angiogenesis and high integrin expression in differentiated MSCs via the Rho signaling pathway. (a) MSCs were cultured with EC differentiation medium in the presence or absence of the Rho inhibitor C3 transferase or the Rho-associated protein kinase (ROCK) inhibitor Y27632 for 4 d and then MTT assay was performed to test cellular viability. (b) MSCs were pretreated with either the C3 or Y27632 in the present of VEGF for 4 d and then continuously cultured in EC differentiated medium for 3 d. Matrigel angiogenesis assays were then performed. (c) Capillary-like structures were quantified by measuring the polygonal network. (d) The expression of integrins *α*1, *α*5 and *β*1 during endothelial differentiation of MSCs was estimated by real-time PCR. ^∗∗^ *P* < 0.01, *n* = 3. (e) Western blotting assay for integrins *α*1, *α*5 and *β*1. (f) The relative quantification of the protein expression. ^∗∗^ *P* < 0.01, *n* = 3.

VEGF has been reported to induce Rho/ROCK activation and promote angiogenesis in ECs [11]. To assess whether VEGF induced angiogenesis in differentiated MSCs via the Rho/ROCK pathway, MSCs were pretreated with either the Rho inhibitor C3 transferase or the Rho-associated protein kinase (ROCK) inhibitor Y27632 in the present of VEGF for 4 d and then continuously cultured in EC differentiated medium for 3 d, and matrigel angiogenesis assays were performed. Cellular viability and proliferation were not inhibited by MTT when MSCs were pretreated with either the C3 or Y27632 for 4 d (Figure 4(a)). Inhibiting Rho/ROCK signaling blocked capillary-tube formation in differentiated cells (Figures 4(b) and 4(c)). As shown in Figures 4(d)–4(f), pretreating cells with 2 *μ*g/mL C3 transferase or 10 *μ*mol/L Y27632 abrogated VEGF-induced integrin *α*1, *α*5 and *β*1 expression in differentiated MSCs. These results suggested that blocking Rho/ROCK signaling could inhibit the upregulation of integrins induced by VEGF and endothelial angiogenesis in MSCs.

### 3.5. Knocking Down Endogenous MRTF-A Obstructed Angiogenesis and Migration in HUVECs by Affecting Integrin Expression

**Figure 5:**
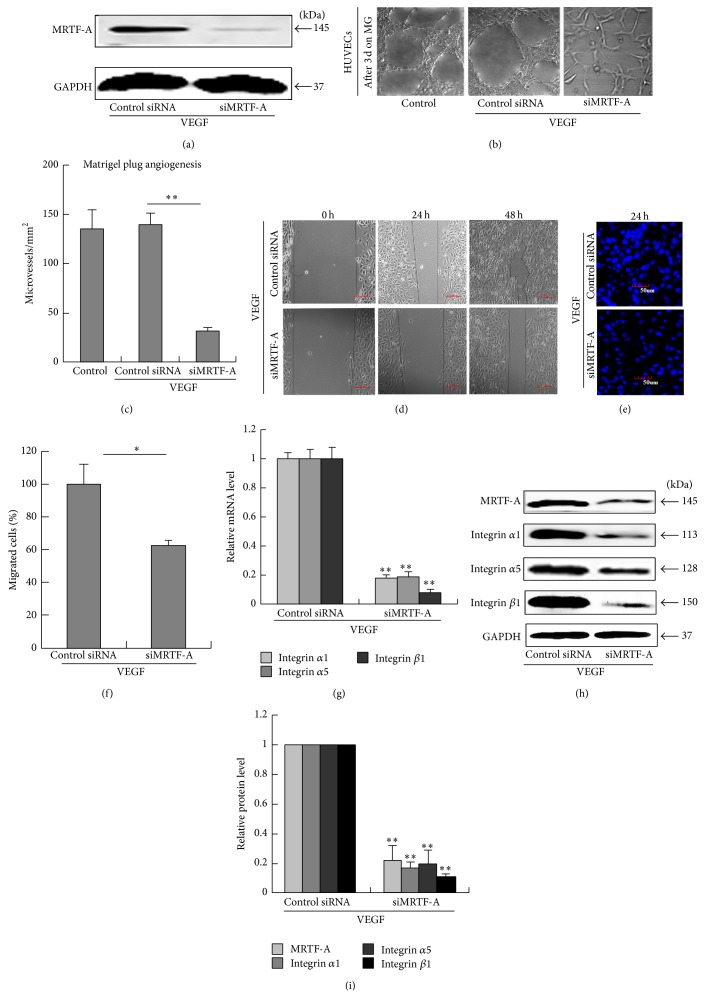
Knocking-down MRTF-A obstructed angiogenesis and migration in HUVECs by affecting integrin expression. (a) Western blotting confirmed the inhibition of endogenous MRTF-A in HUVECs transfected with siMRTF-A. (b) The capillary-like structures formed by HUVECs were observed on Matrigel. (c) Quantification of the capillary-like structures. ^∗∗^ *P* < 0.01, *n* = 3. The migratory ability of HUVECs transfected with siMRTF-A or control siRNA was determined by wound healing (d) and transwell chamber assays (e). (f) Cell migration was quantified by calculating relative cell numbers. ^∗^ *P* < 0.05, *n* = 3. (g) After transfection with siMRTF-A or control siRNA, HUVECs were treated with VEGF for 24 h, and the expression of integrins *α*1, *α*5, and *β*1 was then estimated by real-time PCR. ^∗∗^ *P* < 0.01, *n* = 3. (h) Western blotting for integrins *α*1, *α*5 and *β*1. (i) The relative quantification of the protein expression. ^∗∗^ *P* < 0.01, *n* = 3.

To further confirm that MRTF-A is essential for angiogenesis, we performed siRNA-mediated knockdown experiments in HUVECs. Knocking down endogenous MRTF-A inhibited cell migration and tube formation in HUVECs (Figures 5(a)–5(f)). MRTF-A knockdown led to a marked inhibition of integrin *α*1, *α*5 and *β*1 expression (Figures 5(g)–5(i)). These results confirmed that MRTF-A and its target genes, including integrins *α*1, *α*5, and *β*1, were essential for migration and capillary-like structure formation in HUVECs.

## 4. Discussion

As most of the previously published data regarding the role of MRTF-A were focused on muscle differentiation, development [16], and tumor migration [12], little is known about the functions of MRTF-A in endothelial differentiation, migration, and angiogenesis. The present study identifies MRTF-A as being essential for the migration and angiogenesis of MSC-derived ECs.

Two recent reports investigated the role of SRF and MRTF-A in endothelial cells [17, 18]. Weinl et al. demonstrated that EC-specific loss of either SRF or MRTF-A/MRTF-B impaired filopodia protrusion in endothelial tip cells during postnatal angiogenesis, resulting in incomplete formation of the retinal primary vascular plexus and absence of the deep plexi. Furthermore, MRTF-A and MRTF-B but not ternary complex factor family (TCF), another SRF cofactor, were found to be essential for retinal angiogenesis. Franco et al. reported that knocking down MRTF-A protein levels in HUVECs using specific siRNA led to a significant downregulation of myosin regulatory light polypeptide 9 (MYL9), nonmuscle myosin heavy chain 9 (MYH9) and myosin heavy chain 10 (MYH10) mRNA levels, as well as migratory deficiencies. Notably, we show for the first time that MRTF-A is required for migration and angiogenesis in MSC-derived ECs. Overexpressing sh-MRTF-A significantly destroyed fiber formation, leading to a nearly 60% reduction in the number of microvessel tubes formed.

Some integrins, expressed by endothelial cells, are strongly upregulated on neovasculature, for example, *α*1*β*1 and *α*5*β*1. Previous studies have indicated that the inhibition of integrin *β*1 via antibody- or peptide-based approaches blocks angiogenesis in vivo [19, 20], and the integrin *β*1 family has been shown to play a role in angiogenesis [21]. Integrin *α*5 knock-out mice show embryonic lethality with major vascular defects, including a marked decrease in the complexity of the vasculature and inappropriately formed vessels in both mouse embryos and embryoid bodies [22, 23]. Therefore, the integrin *α*5*β*1 receptor seems to have a definite impact on blood vessel formation and maturation. Here, we observed increases in the expression of integrins *α*1, *α*5 and *β*1 during angiogenesis in MSCs. It has been reported that endothelial VEGF-induced differentiation in human placental multipotent MSCs is accompanied by an increase in cell surface integrin *α*5*β*1 [23]. Treatment with function-blocking antibodies against integrins *α*5 and *β*1 inhibited the formation of capillary-like structures in vitro. Together with previous reports [24–26], these results demonstrate that integrins *α*1, *α*5 and *β*1 are crucial for the formation of new blood vessels by differentiated MSCs.

Furthermore, our results also demonstrated that MRTF-A was required for VEGF-induced integrin expression as well as angiogenesis. When we knocked down MRTF-A by RNA-mediated interference, we observed a striking downregulation of the expression of integrins *α*1, *α*5 and *β*1 during angiogenesis. SRF/MRTF-A was reported to bind to the enhancer element CArG in the genomic locus of integrin *β*1, resulting in cancer cell migration and invasion [27]. Cheli et al. compared integrin *α*1 mRNA levels in megakaryocytic and non-megakaryocytic cells and analyzed the transcriptional activity of a series of integrin *α*1 promoter-luc reporter gene constructs, finding that SRF could bind to the integrin *α*1 promoter and enhance its transcriptional activity [28]. Our data showed that MRTF-A could bind to the SRF-binding site (CArG box) within integrin *α*1 and *α*5 promoters and activate the transcription of integrins *α*1 and *α*5.

Rho signaling is reportedly essential for both VEGF-dependent in vivo angiogenesis and in vitro capillary formation [29–31]. Others have reported that inhibiting the Rho pathway disrupts VEGF-mediated EC activation based on in vivo angiogenesis and in vitro tube-formation assays [4]. In this report, we explored whether the Rho/ROCK signaling pathway plays an essential role in angiogenesis in MSCs. We have shown that treatment with either the Rho inhibitor C3 transferase or the Rho-associated protein kinase (ROCK) inhibitor Y27632 completely abrogated vessel outgrowth from MSC-derived ECs, suggesting that Rho/ROCK signaling is essential for MSCs to form vascular networks. Moreover, activating Rho/ROCK signaling can promote MRTF-A nuclear translocation and integrin activation. Similarly, inhibiting Rho/ROCK signaling with C3 transferase or Y-27632 remarkably reduced the expression of specific integrins.

Endothelial differentiation and angiogenesis correlate with Rho/MRTF-A-mediated cytoskeletal alterations and integrin expression. Our results are the first to suggest that Rho/MRTF-A-mediated integrin expression is involved in numerous aspects of the angiogenic process of MSCs and that this is essential for VEGF-mediated endothelial differentiation, migration and angiogenesis. However, MRTF-A, much like SRF, is known to have multiple functions, including roles in cardiomyocyte, smooth muscle differentiation and development. Nobusue et al. suggested that the regulation of MRTF-A drove MSC differentiation into adipocytes by actin cytoskeleton dynamics [32]. MRTF-A is also reported to be involved in smooth muscle differentiation of MSCs. MSCs have the potential to differentiation along SMC lines in response to Rho/ROCK signaling activation, as evidence by a cytoskeletal protein SM-*α*-actin filament polymerization [31]. Together with our results, MRTF-A is an important transcription factor that regulates MSC differentiation in general by activating specific marker gene expression, or by altering cytoskeleton structures. The endothelial-specific functions of MRTF-A are still poorly understood. Therefore, functional analyses of both MRTF-A and MRTF-B, including endothelial differentiation, survival, migration and the maintenance of vascular integrity and function, should be addressed in the future to illuminate the contributions of these factors to physiological vasculogenesis and angiogenesis and pathological vascular remodeling.
